# Definitions of *Culturally and Linguistically Diverse (CALD)*: A Literature Review of Epidemiological Research in Australia

**DOI:** 10.3390/ijerph18020737

**Published:** 2021-01-16

**Authors:** Thi Thu Le Pham, Janneke Berecki-Gisolf, Angela Clapperton, Kerry S. O’Brien, Sara Liu, Katharine Gibson

**Affiliations:** 1 Monash University Accident Research Centre, Monash University, 3800 Clayton, Australia; Janneke.Berecki-Gisolf@monash.edu (J.B.-G.); Angela.Clapperton@unimelb.edu.au (A.C.); Sara.Liu@monash.edu (S.L.); 2 Centre for Mental Health, Melbourne School of Population and Global Health, Faculty of Medicine, Dentistry and Health Sciences, The University of Melbourne, 3010 Melbourne, Australia; 3 School of Social Sciences, Monash University, 3000 Melbourne, Australia; 4 Department of Health & Human Services, 3000 Melbourne, Australia; Katharine.L.Gibson@dhhs.vic.gov.au

**Keywords:** review, epidemiological, Culturally and Linguistically Diverse (CALD), aboriginal, Torres Strait Islander, indigenous peoples, CALD, migrant

## Abstract

*Objective*: To identify how Culturally and Linguistically Diverse (CALD) communities are defined in epidemiological research in Australia and provide a definition of CALD status that aids the consistency and interpretability of epidemiological studies. *Methods*: Peer-reviewed literature from January 2015 to May 2020 was searched via four databases (Ovid Medline combined with PubMed, Embase, Emcare, and CINAHL) to identify quantitative studies of CALD people in Australia. *Results*: A total of 108 studies met the criteria for inclusion in the review. Country of birth was the most commonly used CALD definition (*n* = 33, 30.6%), with combinations of two or more components also frequently used (*n* = 31, 28.7%). No studies used all the components suggested as core to defining CALD status. including country of birth, languages other than English spoken at home, English proficiency, and indigenous status. *Conclusions*: There was considerable inconsistency in how CALD status was defined. The review suggests that CALD status would best be defined as people born in non-English speaking countries, and/or who do not speak English at home. Additionally, indigenous peoples should be considered separately. This recommended definition will support the better identification of potential health disparity and needs in CALD and indigenous communities.

## 1. Background

Australia is a multicultural society experiencing rapid immigration from diverse countries, and, accordingly, the cultural and linguistic diversity of Australia has increased. The 2016 census showed that more than 28% of the Australian population was born overseas [1]. Recent figures from the Australian Bureau of Statistics (ABS) revealed that over 300 separate ethnic backgrounds were identified in the 2016 census, with over 300 separately identified languages spoken in Australian homes [2]. In 2016, one-fifth of Australians spoke a language other than English at home [2].

Indigenous peoples were the first Australians and the original inhabitants of the land, who also contributed an important part of the cultural and linguistic diversity of Australia. While different terms have been used to refer to indigenous peoples, such as Aboriginal and Torres Strait Islander peoples, indigenous Australians/peoples, Australia’s indigenous peoples, first people/nations/Australians, and traditional owners, we have decided to use the term “Indigenous peoples/individuals” throughout this study. This term is used by the United Nations in the “United Nations Declaration on the Rights of Indigenous peoples” [3]. In 2016, an estimated 798,365 indigenous peoples were living in Australia, representing 3.3% of the total Australian population [4]. More than 250 indigenous Australian languages, including 800 dialectal varieties, were spoken. Among indigenous peoples, 11% (46,700 people) spoke an indigenous Australian language as their main language at home [5]. These statistics highlight the fact that Australia has over seven million people (of 25 million) who may be defined as being from Culturally and Linguistically Diverse (CALD) backgrounds, including migrants or individuals of indigenous descent.

Due to the large number of Australians belonging to CALD communities, research into cultural diversity has been attracting significant attention from researchers and policy-makers in Australia. These groups of people have been reported to experience health disparities [6,7,8,9] and unique conditions such as language barriers, prejudice, discrimination, and racism. An inquiry from the Australian government in 2013 found that new migrants with lower levels of English proficiency and indigenous peoples were statistically more likely to be overrepresented in social-economic disadvantaged groups [10] due to relatively high rates of unemployment and limited access to available services [11]. Importantly, taking cultural differences and accessibility into account when developing interventions can help enable resources to be accessible, inclusive, and responsive to the needs of all people who require assistance. In contrast, poor identification and communication with CALD populations might lead to ineffective resource allocation and interventions. Therefore, research into cultural and linguistical diversity is recommended in seeking to address health and social inequities [12,13].

In order to conduct research about people from multicultural communities, defining the CALD status of study participants is the first important step. While the term CALD is commonly used in Australia, the acronym CALD might be replaced by other terms, such as ethnicity (widely used in England); BAME (Black and Asia Minor Ethnicity); BAEM (Black and Asia Ethnic Minority); minority ethnic groups; race; and specific names of cultural backgrounds such as African, Asian, and Hispanic in an international context. The term “Cultural and Linguistic Diversity”, introduced by the ABS in 1999, is a broad concept drawing attention to both the linguistic and cultural characteristics of multicultural populations living in Australia [14]. To measure the CALD status of individuals in data collection, the ABS (1999) recommended a standard set of cultural and language diversity measures to be used to provide the basis of comparability of information [14]. The set includes four primary indicators: “Country of Birth”, “Main Language Other than English Spoken at Home” (herein referred to as “language spoken at home”), “Proficiency in Spoken English”, and “Indigenous Status”. This is the minimum core set that needs to be collected to determine an individual’s CALD status [14]. They also suggest a range of further optional variables that can be added to meet particular information needs and to get more insight into a person’s diversity characteristics. These include “Ancestry”, “Country of Birth of Father”, “Country of Birth of Mother”, “First Language Spoken”, “Languages Spoken at Home”, “Main Language Spoken at Home”, “Religious Affiliation”, and “Year of Arrival in Australia” [14].

Published by the ABS in 2019, the core set is recommended in all administrative and service provision settings where information on CALD is required. However, it is acceptable to omit variables which may not be appropriate or useful [14]; for example, indigenous status can be omitted when researching migrant issues only. This approach can prove to be problematic and can result in various definitions of CALD being used interchangeably in epidemiological studies. As a consequence, there is variation in the reported prevalence of health outcomes and other factors identified in CALD communities. The inconsistency in the definitions of the CALD status of study participants can undermine the comparability and generalisability of research findings [15], consequently, limiting the quality and applicability of such studies.

Therefore, it is necessary to provide a working definition of CALD in order to increase the consistency of defining CALD status in future epidemiological research. The literature review firstly aims to identify the most commonly used definitions of CALD in current quantitative research in Australia. Additionally, we outline and discuss the advantages and disadvantages of the various CALD definitions in research settings, then provide a practical CALD definition for future research, contributing to improving the quality of CALD-related studies in terms of comparability and generalisability.

## 2. Methods

### 2.1. Data Sources

Systematic searches of published research were carried out to capture studies of CALD people in Australia. The search was performed in the databases including Ovid Medline and PubMed combined, Embase, Emcare, and CINAHL (the Cumulative Index of Nursing and Allied Health Literature) in May 2020. These databases were selected because their scopes are biomedicine and health, broadly defined to encompass those areas of the life sciences and behavioural sciences needed by health professionals and others engaged in basic research and clinical care, public health, health policy development, or related educational activities.

### 2.2. Key Terms and Search Strategies

- 1: CALD OR (cultural* and linguistic* divers*) OR cultural* divers* OR multicultural OR multi-cultural: to capture all possible research articles relating to CALD people.
- 2: Australia* OR Victoria* OR New South Wales OR Northern Territory OR Tasmania* OR Queensland: to capture studies conducted in Australia.
- 3: (1) and (2): to capture studies of CALD communities in Australia.

### 2.3. Inclusion Criteria

Studies were included if they used a quantitative research method and mentioned how the CALD or multicultural status of study participants was defined. We based this on the ABS’s suggestion about the components of CALD status, including country of birth, ancestry, religious affiliation, year of arrival in Australia, proficiency in spoken English, and main language other than English spoken at home [14]. As a result, the inclusion criteria were broadened to include refugees, immigrants, and indigenous status and indigenous peoples if they were captured by the search and met other eligibility criteria. Under the concept of the definitions of CALD status of the study participants, we only included articles about indigenous peoples that explicitly mentioned (1) the aspect of cultural diversity and multicultural background of participants and (2) how to identify their indigenous status within the manuscripts (any other terms referring to indigenous peoples accepted).

We limited the results to studies conducted in Australia only. Studies in multiple countries were not considered because of the differences in setting, and because CALD is a term not commonly used outside of Australian literature. We included only peer-reviewed articles in the English language and with full text available (records of conference abstracts were excluded). We decided to limit the searches to articles published in 2015 or later because we are empirically confident that the research results were saturated within the 108 articles included (please refer to Section 3 for more detail). Specifically, we found similar definitions of CALD repeatedly, and going back further did not add any types of studies that were not already captured and therefore did not provide further insights into CALD studies/terminology.

### 2.4. Data Selection Process

Only peer-reviewed quantitative studies indicating the definition of CALD status of study participants were included in the present review.

**Figure 1:**
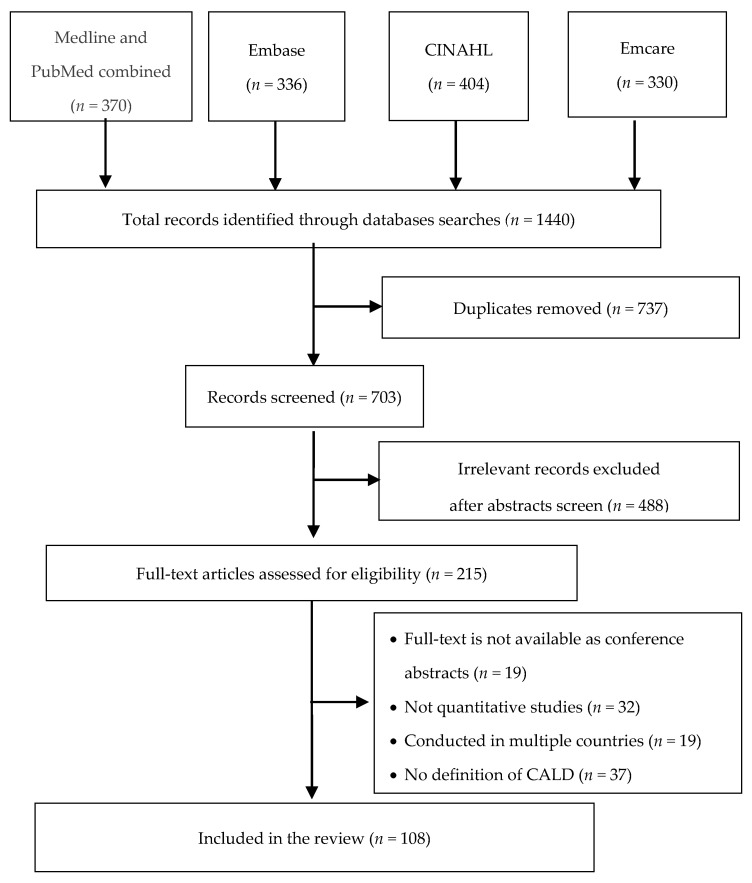
Summary of search and review process.

Literature searches were conducted (Figure 1) with four selected databases. In the first step, we deleted duplicate article results. Titles and abstracts were then screened in accordance with the inclusion and exclusion criteria. Finally, full-text articles were critically examined to find relevant studies for data analysis.

### 2.5. Data Extraction and Analysis

Information regarding the definitions of CALD status, research topics, research locations, study design, data sources, and potential pros and cons of each CALD definition were gathered into an excel template. The data were then coded and analysed to determine the number of articles and percentages within each defined category based on CALD definitions and by the sources of data used. The limitations and strengths of the CALD definitions were extracted from the selected studies. These were then discussed in the context of CALD definitions more generally (Australian and international; not limited to studies included in the review) to compare the pros and cons of each definition and to suggest the most applicable definition of CALD status for research purposes.

## 3. Results

The systematic literature searches revealed 1440 eligible papers (Figure 1), of which 737 duplicates were identified and deleted. Titles and abstracts were then examined in accordance with the inclusion and exclusion criteria. After this screening, we excluded 488 articles because they were not quantitative studies (according to the research methods mentioned in abstracts) or failed to recruit or include CALD people as participants. We then reviewed the full-text versions of the remaining 215 publications to further evaluate them for eligibility: 19 articles were excluded because they were conference abstracts (first/corresponding authors were contacted). After evaluating the remaining 196 full-text articles, 37 articles were excluded because they did not report a definition of CALD status, 19 studies were excluded due to multiple-country study sites, and 32 because they were not quantitative studies. These studies did not mention all relevant information regarding research methods in the abstracts, but we found them to be non-quantitative research after full-text reading. The screening process resulted in 108 articles meeting the inclusion requirements and being included in the review.

The 108 selected epidemiological studies covered a variety of topics such as aged care, antenatal care, asthma, and autism (Table 1). The majority of the studies examined CALD communities in New South Wales (*n* = 41), followed by Victoria (*n* = 29), Queensland (*n* = 13), Western Australia (*n* = 4), South Australia (*n* = 3), and Northern Territory (*n* = 1). Another 15 studies examined CALD groups in the whole of Australia or without mentioning the specific locations of CALD participants, whilst two other studies looked at three specific Australian states—namely, New South Wales/Victoria/Western Australia and Victoria/New South Wales/Queensland.

Regarding the data sources and study designs of the selected studies, approximately half of them used available data (*n* =52.5%), including administrative hospital databases, household surveys, and data collected in other studies. The remainder collected data via face-to-face interviews, self-reported surveys, or phone calls (*n* = 56.5%). There was considerable variability in how the study designs were described in these articles: some categorised themselves as descriptive, quantitative, experimental, observational, or mixed qualitative and quantitative studies; others mentioned the exact terminology of their research methods. Specifically, the most common (self-reported) study designs/types were cross-sectional studies (*n* = 54), followed by 17 retrospective studies and 5 longitudinal studies (Table 1).

Among 108 articles, 88 papers explicitly mentioned CALD definitions in the methodology (81.5%), whilst the remaining articles did not explicitly state how CALD status was defined, but this information could be inferred from the manuscripts.

### 3.1. Definitions of CALD

Table 1 presents how CALD status was defined in each article, demonstrating that CALD definitions were inconsistently used in the selected 108 quantitative studies. The various definitions of CALD from these studies were categorised into six main groups, as shown in Table 2.

Although country of birth (COB), language spoken at home (LSAH), English proficiency, and indigenous status make up the minimum core set to measure CALD status suggested by The Australian Bureau of Statistics [14], Table 2 shows that no articles used all four, with English proficiency not reported by any of the studies included in this review. COB, LSAH, and indigenous status were each commonly used as a stand-alone definition of CALD, in which COB was the most frequently used definition (used in 33 articles: 30.6%), LSAH was used in 21 articles (19.4%), and indigenous status was used in 3 articles (2.8%). The following are the detailed measurements of these three definitions:

“Country of birth” was used in 33 articles. There were two methods to define CALD status of participants by COB. First, if the respondents reported a COB other than Australia, they were defined as CALD participants, whilst non-CALD participants were those who were born in Australia. This type of definition was used in 16 articles. Second, if the respondents reported non-English speaking countries as COB, they were defined as CALD participants, in contrast to those born in English-speaking countries, who were defined as non-CALD participants. Further, some articles used a particular COB, such as “China” or “Spanish speaking countries”, as the definition (see Table 1).

“Language spoken at home”: in 21 studies, the participants were asked about the language they used in daily conversation at home. If it was English, they were categorised as being in non-CALD groups, whereas non-English speakers were categorised as being in CALD groups.

“Indigenous status”: only three articles that used indigenous status as a measure of CALD were captured by the current literature review’s search strategies. There could be other articles that used similar methods to define CALD but that did not meet the search criteria (i.e., explicitly mentioning cultural diversity and multicultural), and hence failed to be captured by the present literature review. In these articles, indigenous status was self-reported by answering whether the participants were born to indigenous mothers or not (in 1/3 studies). Additionally, they were categorised as CALD if they responded “Yes” to the question regarding indigenous status (1/3 studies) or Māori and Pacifica Islander background (1/3) (see Table 1).

In addition to the use of the variables in the minimum core set to define CALD status, “ethnicity”, “cultural”, or “CALD background” were also used as CALD definitions in 13.9% of the selected articles (*n* = 15). Overall, approximately 30% of the articles clearly stated how they measured ethnicity/cultural background, whilst the rest had no information (Table 1). Among the studies that provided inadequate information on the measurement of ethnicity (cultural background/CALD), some authors possibly used an ethnicity (cultural background/CALD) variable readily available from administrative or secondary datasets, failing to mention how it was collected. CALD backgrounds were also self-reported by participants reflecting how they perceived their CALD/ethnicity status; again, instruction on how people self-evaluated CALD background was missing in the manuscripts. Although some authors stated how ethnicity (or cultural background) was measured, no consistent measurement of ethnicity was found. For example, in one study, ethnicity was defined through self-reported nationality, in which people were classified as CALD if they self-identified as having a nationality or mix of nationalities other than Australian. In contrast, in another study, the participants’ ethnicity was based on their own culture, as well as their language and beliefs, rather than strictly on nationality or country of birth. Further, participants were recruited from specific community groups with Chinese and Vietnamese backgrounds in other studies. To summarise, there was variation in defining ethnicity, with some studies inadequately reporting their definition. This further contributed to CALD status being measured differently across studies.

Furthermore, “migrant” and “refugee” statuses were used in five articles (4.6%) based on people’s self-report of having migrated from another country, or were identified by visa types.

Definitions using combinations of more than one component to categorize CALD or non-CALD status were the second most common method of defining CALD status (31 articles: 28.7%), following the definition by COB only. This measure used two or more indicators of CALD status; for example, when COB and LSAH were combined, participants with CALD status were those born in non-English speaking countries and/or those who spoke a language other than English at home. More examples of these definitions such as interpreter required or duration of time staying in Australia are provided in Table 1.

### 3.2. CALD Definitions in Different Data Sources

Although various sources of data were used in the 108 selected articles, COB, LSAH, and the combinations of two or more CALD variables were widely used to define the CALD status of participants. Table 3 presents definitions of CALD per two main data sources: administrative data or other secondary data analysis and self-collected data. Researchers who used administrative or other types of available databases such as national surveys, hospital records, or routine clinical services data were more likely to select combined definitions (36.5%) and COB (34.6%). On the other hand, LSAH (23.2%), ethnicity group (16.1%), migrants and refugees, or indigenous peoples were more common in survey data collected by telephone, face-to-face interviews, or online surveys.

## 4. Discussion

To our knowledge, this is the first literature review to systematically explore how researchers have defined CALD communities in quantitative studies in Australia. The review showed that various definitions of CALD have been inconsistently used in the 108 selected articles, and none of the selected articles used the full minimum core set that is recommended by the ABS [14] to define CALD status.

### 4.1. Pros and Cons of Definitions

Researchers use various definitions to determine the CALD status of their study participants; this is likely due to the flexible guidelines for defining CALD by its components suggested by the ABS [14]. Each definition has its advantages and disadvantages, which can determine suitability depending on data availability and difficulties in data collection. The inconsistency in using a variety of CALD definitions might limit the generalisability of study results and make them difficult to compare. Therefore, this review critically analysed the strengths and limitations (of each definition) which were pointed out by published literature (not limited to articles included in the review) from Australia and other countries to broadly understand how CALD participants were identified, then to provide an evidence-based suggestion for how future research could define CALD status to collect data related to CALD communities.

### 4.2. Country of Birth

One third of the selected articles used COB to define CALD status; this was the most widely used definition (*n* = 33, 30.6%). This is consistent with the CALD definition in the chapter of CALD population health under “Australia’s Health 2018” reported by the Australian Institute of Health and Welfare (AIHW) [123]. AIHW stated that defining the CALD population by COB was used in the report as it was the most common data element among AIHW health data collections [123]. Additionally, this definition was commonly used as a proxy measure for CALD, particularly in clinical settings, as it indicates individuals’ geographical place of origin and is consistently presented over time.

While some argue for the benefits of using country of birth due to the ease of data collection and consistency in presenting [124], others have identified limitations to this definitional approach. First, there appears to be variation in reporting and recording these data [125,126]. Tran et al. (2012) found that 19% of overseas-born people in New South Wales Admitted Patient Data Collection incorrectly reported their COB [125]. The authors mentioned that COB was mainly recorded inaccurately as “Australia” or “Inadequately described”, and that it was recorded with greater accuracy in principal referral hospitals, public hospitals, and hospitals in metropolitan areas. Longer duration of stays in Australia deceased the accuracy of COB recorded, and the accuracy increased with age at immigration and varied according to COB [125]. Another study about the accuracy of information provided by pregnant women to survey questions on cultural backgrounds and country of birth supported this notion, as the COB variable was not a reliable proxy measure of cultural background [121]. Second, defining participants who were not born in Australia as CALD means that the CALD group included both people with English-speaking and non-English-speaking backgrounds. This may affect the results, as no linguistic aspects of CALD are considered. As a result, CALD status defined by having a non-English speaking country of birth is likely a better option. Third, CALD defined based on country of birth only might not capture people who were born in Australia but whose families were originally migrants. The reverse may also be true for people who were born overseas but arrived in Australia when they were very young and grew up in Australia. These people could be very different from those who arrive later in their childhood or as an adult, despite all being born overseas. Last, when this definition is applied to indigenous peoples who were born in Australia, they would be categorised as part of the non-CALD population. However, the cultural and linguistic aspects and needs of indigenous peoples are significantly different from those of Anglo-Australians. Thus, it can be concluded that relying on COB only might lead to some misclassifications of CALD because of the failure to capture linguistic and ethnic aspects. Therefore, where possible COB should be combined with other information to define CALD status.

### 4.3. Language Spoken at Home

In the 108 selected articles, 21 articles used LSAH as the definition of CALD. Such studies are likely to explore language barriers, which is one of the most important challenges for CALD people. A limitation of defining CALD by LSAH is that it might be affected by acculturation [58]. Specifically, children of immigrants (from CALD backgrounds) living in Australia for many years might report English as LSAH [52]; therefore, they would be misclassified as the non-CALD group. Further, CALD backgrounds of English-speaking people such as Singaporeans or Indians might also be miscategorized as the non-CALD group by the current definition [52]. Finally, this definition is likely to under-represent the full CALD population and may represent a relatively more culturally isolated subgroup [51]. People who are not English native speakers have a relatively higher level of need for assistance than other subgroups [111]. Thus, using LSAH other than English as the only indicator of CALD is an imperfect definition as only the potential language barrier is considered [50]. This leads to a limited generalisability of the findings to other regions or other cultural groups [62]. Therefore, it is recommended that LSAH should not be the only variable to define CALD status.

### 4.4. Indigenous Status

Defining this group under the CALD concept is challenging for CALD research because indigenous peoples are mostly born in Australia and many of them speak an indigenous language (other than English). In the studies captured in this review, the CALD definition was mostly based on COB, so that indigenous peoples were commonly included in non-CALD communities. However, it becomes difficult if researchers use both COB and LSAH to identify CALD status of indigenous Australian-born peoples who do not speak English at home. This results in a conflict because if the definitions of CALD status are based on COB of participants, this group would be included in the non-CALD group; in contrast, if the LSAH were used, the indigenous peoples would then be categorised in the CALD group (due to speaking an indigenous language at home). Therefore, two approaches can be used here. First, researchers could exclude indigenous peoples from CALD studies, explaining that indigenous peoples are not representative of either CALD or non-CALD groups in their research framework [72,105]; and indigenous research could be conducted in targeted studies instead of being included in more general CALD studies. Second, indigenous peoples could be included in CALD research but categorised in a separate group. This latter approach is a suitable method as the experiences and needs of such first nation people are seen as significantly different from the two other groups—i.e., CALD and non-CALD. For example, Stephane et al. (2015) categorised research participants into three groups, including English-speaking, CALD, and indigenous background [118,119], in their studies of young offenders. Similarly, the ABS (2014) discussed the three most common components of CALD (country of birth, language spoken at home, and English proficiency) together, but separately referred to indigenous people [127].

It is important to note when indigenous peoples are categorised as a separate group, the population size needs to be taken into consideration because the population is much smaller than that of CALD and non-CALD groups in Australia. To support this, Adam (2017) revealed that gaining enough numbers of people for statistical analysis in indigenous status research is often a challenge [70]. Thus, indigenous peoples should reasonably be considered as a separate entity with attention to the challenge of sample size for recruitment and also analysis.

### 4.5. English Proficiency

None of the studies included in this review used English proficiency as an indicator for CALD status. Possibly, this is because information regarding the level of English proficiency is not readily available in most administrative datasets. Additionally, it is normally self-assessed (and therefore subjective), which might lead to limitations of data accuracy. Further studies regarding the availability and quality of data about “Proficiency in Spoken English” among people from non-English speaking backgrounds are recommended.

### 4.6. Ethnicity and Cultural Background

Cultural background and ethnicity are reported as ambiguous definitions of CALD as it is self-evaluated and changeable, without explicit criteria. Internationally, the use of the term “ethnicity” in data collection and the subsequent interpretation of people’s cultural diversity is widely debated [121]. In England, an audit of the accuracy of coding in English National Health Services hospital records found that 39–43% of ethnicity data for ethnic minorities was incorrect [128]. A systematic review of self-harm among minority ethnic groups in the United Kingdom found that: while some studies failed to clearly mention how “ethnicity” was defined, it was variously defined in other studies (based on participants’ names, or countries of birth of participants) [15]. In Australia, the ABS, which is responsible for population census data collection, defines ethnicity as a multi-dimensional concept that may include cultural traditions and customs, shared history, geography, language, religion, and/or identification with a minority group [129]. Ethnicity, cultural identity, or cultural background are complex, dynamic, and multi-layered concepts referring to an individual’s sense of identity, which can change over time and across generations [130,131]. This information is not readily available in administrative data [22], and if available it might be collected for different purposes which can lead to potential bias and misunderstanding. Therefore, cultural background and ethnicity should only be considered to define CALD status if they are captured and presented with clear definitions alongside appropriate and well-described data collection strategies.

### 4.7. Definitions Using More than One Component of CALD

There is no single characteristic that should merely be used to define the CALD status of study participants as CALD is a broad concept drawing attention on both cultural and linguistic characteristics of people. For example, if only asking a person’s country of birth, there is a risk that differences within communities such as language barriers are masked; in contrast, if only asking about the main language spoken at home, cultural differences might not be captured. Researching CALD communities requires researchers to touch on multiple relevant aspects of CALD. Consistent with the ABS (1999), this review found that COB, LSAH, and indigenous status are three of the most important aspects of CALD in Australia [14]. Research methods capturing all three key aspects including linguistic, cultural, and ethnic factors are optimal for identifying any barriers or disadvantages that CALD people may experience. Therefore, we suggest that if feasible, the definition of CALD should involve more than one suitable component of CALD.

### 4.8. Other Definitions of CALD

Other less common CALD definitions such as *interpreter required* or *duration of time staying in Australia* were used when language barriers or acculturation aspects of CALD were the study focus. However, they were less common than COB/LSAH/Indigenous status. The literature review revealed that longer duration of stay in Australia [132] and sufficient English proficiency [133] are significant factors affecting how migrants generally acculturate and adapt to the Australian culture and demands. It is self-evaluated, and therefore, people’s self-evaluation of CALD status changes over time depending on how they feel about their degree of acculturation and adaptation to the host culture. The topic of acculturation is not the prime focus of the current research because interpreter required or length of stay in Australia were less commonly chosen to define CALD status of study participants in the selected articles; however, they could be used as additional components to define CALD when necessary.

### 4.9. Comparison with Research in Other Countries

Not all countries used the term CALD; for example, other terms such as “BAEM (Black and Asia Ethnic Minority)”, “ethnicity”, and “minority ethnic groups” are more common to classify cultural backgrounds of people in other countries such as in England, America, and Canada. However, this review was specifically focussed on cultural and linguistic diversity and not specifically on ethnicity, which tends to be the focus of other studies (the United Kingdom and the United States). Therefore, the study findings cannot be directly compared with research from countries where there is more of a focus on ethnicity (some comparisons were made while discussing “ethnicity”). Additionally, to our knowledge to date, research to evaluate how multicultural backgrounds of study participants were classified in other countries is relatively scarce. No literature review on definitions of CALD status (or other terms) of study participants has been published elsewhere, which leads to limited comparison to similar research in other countries. Further research in this area is recommended.

## 5. Limitations of This Study

This review aimed to investigate the definitions of CALD in quantitative research of CALD communities to recommend a suitable definition of CALD for future studies. This review has limitations that need to be acknowledged. First, the review focused on health research only; this might not reflect the overall definition of CALD in other social fields. Second, the systematic searches were limited to papers indexed in four selected databases, and written in English, not including grey literature. Last, comparisons with other international studies were limited not only because of the specific focus of this review (on cultural and linguistic diversity) but also the availability of similar research in other countries, which was discussed in detail in the previous section.

## 6. Recommendations

The flexible and adaptable application of CALD definitions potentially leads to limitations in terms of research generality and comparison. To contribute to the improvement of future research of CALD groups, a clear-cut definition of CALD status for research purposes is recommended.

Ideally, researchers should capture as many CALD components as possible to avoid the limitations of a single definition. Covering multiple aspects of CALD status leads to a broader representation of CALD communities. However, in practice, the decision of researchers on how to define CALD status is usually limited by data availability and resources to conduct surveys. Researchers using administrative data only have access to variables in the data set they are using, whereas researchers collecting their data have different options. Researchers using administrative data should consider data linkage options to obtain the most complete set of CALD variables possible. Conversely, researchers collecting their own data through survey should at least collect data regarding three components of CALD, including COB, LSAH, and indigenous status (plus any additional ones relevant to the study), as recommended by the ABS [14]. Therefore, three components of CALD should be used as the minimum core indicators of CALD while using any sources of data, and if possible other variables such as English proficiency, ethnicity, and length of stay in Australia should also be obtained.

### 6.1. Definition of CALD Status

In order to define the CALD status of research participants, we suggest a definition of CALD communities based on three main components including COB, LSAH, indigenous status:

- ALD group: people who were born in non-English speaking countries and/or English is not the main language spoken at home. English-speaking countries as suggested by the ABS, 2013 include Australia, the United Kingdom (England, Scotland, Wales, Northern Ireland), the Republic of Ireland, New Zealand, Canada, the United States of America, and South Africa) [134].
- Indigenous group: people who are of indigenous status descent, or self-identified as with indigenous status, or accepted by indigenous communities [3,135]. However, in practice, in the general Australian population, the number of Indigenous peoples are significantly smaller than the CALD and non-CALD communities, which may potentially affect the statistical power of studies. More importantly, when using indigenous identification to define a culturally and linguistically diverse group, researchers should undertake ethical research practices and indigenous data sovereignty consistent with the UNDRIP [3].
- Non-CALD group: people who are neither in CALD nor from Indigenous groups.

This suggestion is to define CALD status of participants in data collection for research purpose only. It is not intended to determine whether a person has CALD status or not.

In the scenario where one or two variables are unavailable and there is no option to obtain them (through data linkage or otherwise), the recommended definition could still be considered as the best applicable measurement of CALD study participants by omitting the unavailable variables from the suggested definition. The flexible applications of the definition may create inconsistency in identifying CALD status and should, therefore, be well-described and acknowledged as limitations of the research.

### 6.2. Key Take-Home Messages

- Existing definitions of CALD status for data collection were inconsistently applied in current epidemiological studies, limiting the interpretability and comparability of such research.
- The use of single components of CALD status in defining CALD communities is insufficient to reflect both cultural and language aspects of CALD communities.
- A clear-cut definition and standardised terminology to define CALD status (for research purposes only) will improve the quality of future research in the area; this paper gives recommendations for such a definition.

## 7. Conclusions

In this review of 108 studies, we have confirmed the inconsistency in defining CALD status as well as the lack of a consistent definition and terminology for Australian epidemiological studies. Each of the specific definitions has advantages and disadvantages which might lead to limitations in the comparability and generalisability of such studies. This highlights the need for a consistent and practical general definition of CALD status for research purposes. After reviewing current published studies using various CALD definitions and critically analysing the pros and cons of each, we propose an evidence-based definition, which is that CALD study participants should be defined in research as those who were born in non-English-speaking countries and/or their main language spoken at home is not English. It is not suggested to determine whether a person “is CALD” or not because there is no explicit criterion to define CALD members. Indigenous peoples should be categorised in a separate group. The application of a clear-cut definition with standardized terminology of CALD status for study participants in data collection (only) will improve the quality and consistency of future research findings.

## Figures and Tables

**Table 1:** Definitions of CALD in the selected studies.

Study	Research Topic	Regions	CALD Definition	Study Designs	Data Sources	Sample Size
**Definitions of CALD by COB**						
Sanchez, 2020 [13]	Migrant health	Queensland	Born in Spanish-speaking Latin American countries	Cross-sectional study	Electronic medical records	382
Ponsford, 2020 [16]	Brain injury	Victoria	Born outside of Australia, New Zealand, the United Kingdom, and the United States	Longitudinal study	Head injury outcome study database	206
Heilat, 2019 [17]	Breast cancer	New South Wales (NSW)	Born in Arabic speaking countries	Retrospective audit	Prospectively maintained institutional database	2086
Counted, 2019 [18]	Quality of life	NSW	Born in Africa	Cross-sectional	Survey	261
Green, 2018 [19]	Palliative care	NSW	Born in non-English speaking countries (NESC)	Retrospective audit	Medical record audit data	100
Wechkunanukul (2016) [20]	Chest pain	South Australia	Born in NESC	Cross-sectional	Hospital administrative data	8225
Ghosh, 2017 [21]	Mental health	Western Australia	Born in NESC	Population-based retrospective cohort study	Linked data using Western Australia Data Linkage System	665,523
Basic, 2017 [22]	Age care	NSW	Born in NESC	Cross-sectional	Referral source, medical diagnoses	2180
Khawaja, 2017 [23]	Mental health	Queensland	Born in NESC	Cross-sectional	Survey	221
Law, 2015 [24]	Physical activity	Victoria	Born in NESC	Mixed method	Survey	402
Lin, 2016 [25]	Aging	Victoria	Born in NESC	Cross-sectional	Survey by questionnaire	122
Casey, 2015 [26]	Occupational safety	Australia	Born in NESC	Cross-sectional	Self-evaluation questionnaire	562
Mazza, 2018 [27]	Cancer	Victoria, NSW, Queensland	Born in NESC	Observational cohort study using a mixed-method approach	Survey	60
Trinh, 2017 [28]	Caesarean section	NSW	Born overseas	Observational study	Administrative hospital data	48,711
Tilley, 2015 [29]	HIV/AIDS	Australia	Born overseas	Observational clinical cohort study	Australian HIV Observational Database	3894
Ogbo, 2019 [30]	Depression	NSW	Born overseas	Retrospective study	Linked retrospective maternal and child health data	20,560
Ogbo, 2019 [31]	Breastfeeding	NSW	Born overseas	Retrospective study	Linked retrospective maternal and child health data	25,407
Berry, 2018 [32]	Neuropsychology	Australia	Born overseas	Cross-sectional	Normative data obtained from Chinese Australian Neuropsychological Normative Study	145
Wallace, 2018 [33]	Neuropsychology	Australia	Born overseas	Cross-sectional	Normative data obtained from Chinese Australians Neuropsychological Normative Study	145
Dahlen, 2018 [34]	Violence and perinatal outcomes	NSW	Born overseas	Retrospective population-based data study	Western Sydney Local Health District Obstetric database	33,542
Maneze, (2016) [35]	Survey responses	NSW	Born overseas	Cross-sectional	Online Survey	552
Harrison, 2016 [36]	Sexual health	NSW	Born overseas	Pilot study	Survey	262
Glew, 2015 [37]	Nursing students	NSW	Born overseas	Prospective study	Survey	2669
Poon, 2015 [38]	Psychosis	Victoria	Born overseas	Longitudinal study	Survey	52
Tirlea, 2016 [39]	Improve self-esteem	Victoria	Born overseas	Cluster randomized control trials	Survey	122
Kisely and Xiao, 2018 [40]	Mental health	Western Australia	Born outside of Australia, New Zealand or the British Isles	Case-control	Administrative health data	5916
Tervonen, 2017 [41]	Cancer	NSW	Born in NESC	Cohort study	Population-based NSW Cancer Registry	651,245
Thai, 2017 [42]	Cancer	Victoria	Born in NESC	Retrospective analysis	Survey	143
Bhaskar, 2019 [43]	Stroke	NSW	Born overseas	Retrospective study design	Administrative datasets	3537
MacLachlan, 2018 [44]	Hepatitis b	Australia	Born overseas	Cross-sectional study	Linkage data of Census and seroprevalence data	237,894
Xiao, 2020 [45]	Aged care	South Australia	Born overseas	Pre-and post-evaluation design	Survey	113
Strugnell, 2015 [46]	Physical activity	Victoria	Participants or parents were born in NESC	Cross-sectional study	Survey, self-reported	286
Gibbs, 2015 [47]	Dental health	Victoria	Born overseas	Exploratory trial	Survey	692
**Definitions of CALD by LSAH**						
Kilkenny, 2018 [48]	Stroke	Australia	Interpreter required/language barrier -as LSAH other than English	Observational Study	Australian stroke Clinical Registry	1461
Bent, 2015 [49]	Autism	Australia	LSAH other than English	Cross-sectional study	Available database	15,074
Scott, 2019 [50]	Obesity	Victoria	LSAH other than English	Cross-sectional study	Data of children participating in Healthy Together Victoria and Childhood Obesity Study	2407
Guo, 2019 [51]	Discharge against medical advice	NSW	LSAH other than English	Cross-sectional study	Hospital records	192,948
Hardy, 2018 [52]	Obesity	NSW	LSAH other than English	Cross-sectional study	Population health Survey	26,449
Beauchamp, 2020 [53]	Breast cancer screening	Victoria	LSAH other than English	Randomised controlled trials	Surveyed by Breast Screen Victoria	1032
Cullerto, 2016 [54]	Cancer	Queensland	LSAH other than English	Pilot program/intervention study	Collected via quantitative interviewer-administered questionnaire	146
Emerson, 2019 [55]	Rehabilitation	NSW	LSAH other than English	Longitudinal study	Interview	102
Okely, 2017 [56]	Physical activity	NSW	LSAH other than English	Cluster non-randomized trial	Survey	420
Soon, 2018 [57]	Cancer	NSW	LSAH other than English	Cross-sectional	Survey	168
Zhang, 2018 [58]	Cardiac rehabilitation	NSW	LSAH other than English	Descriptive, case-matched comparative study	Survey	90
Martin, 2016 [59]	Education	NSW, Victoria, and Western Australia	LSAH other than English	Cross-sectional study	Survey	450
Abu-Arab, 2015 [60]	Challenges to supervise cald nursing students	Victoria	LSAH other than English	Cross-sectional study	Survey	19
Barnett, 2015 [61]	Physical activity	Victoria	LSAH other than English	Cross-sectional study	Survey	261
O’Hara, 2018 [62]	Cancer	Victoria	LSAH other than English	Cross-sectional study	Survey	317
Freyne, 2018 [63]	Ehealth allied health	Victoria	LSAH other than English	Cross-sectional study	Survey	45
Arjunan, 2019 [64]	Body index	NSW	LSAH other than English	Cross-sectional study	Survey	272
Lim, 2019 [65]	Cancer	NSW	LSAH other than English	Cross-sectional study	Paper-based questionnaire	68
Foster, 2018 [66]	Sex industry	NSW	LSAH is Chinese and Thai	Cross-sectional study	Self-completed questionnaire from previous survey	435
Cranney, 2018 [67]	Chronic disease	NSW	LSAH is Mandarin or Cantonese	Mixed methods	Survey (self-administered structured questionnaire)	253
Wood, 2015 [68]	Asthma	NSW	LSAH other than English	Cross-sectional nested study	Survey	144
**Definitions of CALD by indigenous status**						
Mihrshahi, 2017 [69]	Eating and physical activity	Queensland	Indigenous status	Quantitative uncontrolled pre-post design	Survey	375
Adams, 2017 [70]	Diabetes	Victoria	Indigenous status	Mixed-methods study design	Survey	129
Davison, 2018 [71]	Stress	North Territory	Indigenous people	Cross-sectional study	Data from the Life Course Program	576
**Definitions of CALD by ethnicity/cultural background or CALD status**						
Dudley, 2015 [72]	Physical activity	NSW	Asian or Middle-Eastern cultural backgrounds, indigenous people excluded	Prospective cohort study	School enrolment records	658
Wickramasinghe, 2019 [73]	Paediatric care	NSW	Cultural background	Retrospective audit	Hospital records	279
Eh, 2016 [74]	Diabetes	NSW	Cultural background (Chinese background)	Cross-sectional study	Self-completed questionnaire	139
Masri, 2019 [75]	Chronic disease	NSW	Ethnicity	Cross-sectional study	Data collected through the 45 and Up Study	41,940
Lennox, 2017 [76]	Oral language proficiency	Queensland	Ethnicity	Broader longitudinal study	Students’ assessment data, Australian Early Development Census	104
Lau, 2016 [77]	Simulation training	Victoria	Ethnicity	Pilot study used mixed methods	Survey	45
Gallegos, 2020 [78]	Chronic disease	Queensland	Ethnicity	pragmatic evaluation	Face-to-face data collection	700
Burns, 2018 [79]	Iodine deficiency	NSW	Ethnicity	Cross-sectional study	Survey	97
Gallegos, 2019 [80]	Cardiometabolic risk	Queensland	Ethnicity	Cross-sectional study	Survey	693
Bloomer, 2019 [81]	End-of-life care	Victoria	Ethnicity and religion	Retrospective study	Medical record audit	132
Gallegos, 2019 [82]	Physical activity	Queensland	Ethnicity, time in Australia	Cross-sectional	Routinely collected clinical information	700
Khawaja and Ramirez, 2019 [83]	Acculturation	Australia	Self-defined CALD status	Non-experimental	Survey	229
Wantanabe, 2017 [84]	Age care	NSW	Self-determined ethnicity.	Mixed methods	Surveyed by postal questionnaire	82
Hum and Carr, 2018 [85]	Gambling	Victoria	Self-reported cultural background	Cross-sectional	Survey	628
Singh, 2019 [86]	Chronic disease	Victoria	Self-reported Indian ethnicity	Cross-sectional	Paper Survey	138
**Definitions of CALD by migrant/refugee**						
Correa-Velez, 2015 [87]	Wellbeing of refugee	Australia	Migrant	Mixed method, longitudinal analysis	Survey	120
Ploot, 2018 [88]	Psychological	Australia	Migrant	Cross-sectional study	Online Survey	1334
Satyen, 2018 [89]	Family violence	Australia	Migrant	Cross-sectional study	Survey (self-administered)	130
Khawaja, 2018 [90]	Acculturation	Queensland	Migrant and refugee	Cross-sectional study	A part of a larger study	237
Specker, 2018 [91]	Post-traumatic stress disorder	Australia	Refugee or asylum-seeker background	Cross-sectional study	Online questionnaire	246
**Definitions of CALD by combining two or more components**						
Waller, 2020 [92]	Organ donors	NSW	Born overseas/non-English speaking	Cohort study	Linkage data using available databases	2977
Eapen, 2017 [93]	Child development	NSW	Born overseas/non-English speaking	Longitudinal birth cohort study	Routinely clinical data collection	1763
Rowe, 2017 [94]	Injecting drug use	NSW	Born outside Australia/non-English speaking, self-defined cultural background	Cross-sectional	Survey	560
Basu, 2019 [95]	Mental health	Victoria	Born overseas/parents were born overseas/from a non-English-speaking or Aboriginal background	Retrospective study	Survey	101
Cyril, 2017 [96]	Obesity	Victoria	Born overseas, spoke a language other than English at home	Cross-sectional study	Survey	39
Krishnaswamy, 2018 [97]	Antenatal vaccination	Victoria	Born overseas, first language was not English	Cross-sectional study	Survey	537
Martin, 2019 [98]	Cultural identification	Australia	COB and cultural backgrounds/ethnicity	Quantitative studies	Survey	133
Gemert, 2016 [99]	Hepatitis b test	Victoria	COB and ethnicity, indigenous peoples	Non-randomised, pre-post pilot study	Clinic electronic health records	33,297
Rowe, 2020 [100]	Drug, alcohol; tobacco	Australia	COB and/or language	Cross-sectional study	The 2013 National Drug Strategy Household Survey	22,696
Moss, 2019 [101]	Mental illness	Queensland	Born in non-English speaking countries, interpreter required;	Cross-sectional study	the Transitions of Care and the Patient Integrated Mental Health Application	976
Mozooni, 2020 [102]	Stillbirth	Western Australia	Born overseas/ethnicity (indigenous births excluded)	Retrospective cohort study	Linked administrative health data	260,997
Nicholson, 2016 [103]	Early home learning	Victoria	Born overseas/LSAH other than English	Cluster randomised controlled trial	Survey	1200
Jessup, 2017 [104]	Health literacy	Victoria	Born in NESC, LSAH other than English	Cross-sectional study	Self-reported written Survey	384
Mander and Miller, 2016 [105]	Maternity care	Queensland	Born in NESC, LSAH other than English	Cross-sectional study	Available data from Having a baby in Queensland Survey	4704
Brady, 2018 [106]	Chronic disease	NSW	Born in NESC, LSAH, interpreter required, self-identified ethnocultural	Pilot randomised controlled trial.	Survey	48
Blay, 2018 [107]	Interpreter utilisation	NSW	Born in NESC, LSAH other than English	Retrospective analysis	Health administrative data	19,627
Ghayour-Minaie, 2019 [108]	Substance use	Victoria	Born overseas/LSAH is not English	Cross-sectional study	Obtained as part of the large-scale “Resilient Families Research Initiative”	2080
Attrill, 2017 [109]	Speech–language pathology	South Australia	Born overseas/LSAH is not English	Cross-sectional study	Survey	854
Kisely, 2020 [110]	Community treatment orders	Queensland	Born outside of Australia, NZ, UK, Ireland, and North America, or preferred language is not English/indigenous	Cases and controls	Linked administrative health data	7432
Zhou, 2016 [111]	Disability	Australia	Born overseas/LSAH is not English	Cross-sectional study	Two ABS collections of data	312,539
Ogbo, 2016 [112]	Breastfeeding	NSW	COB, LSAH, ethnic, dress, traditions, food	Retrospective clinical audit	Routinely collected perinatal data	17,564
Ogbo, 2018 [113]	Antennal depression	NSW	COB, LSAH, ethnic, dress, traditions, food	Retrospective clinical audit	Routinely collected perinatal data	17,564
Hwang, 2018 [114]	Cancer	NSW	COB, LSAH, interpreter required	Retrospective study	Clinical Cancer Registry	1215
James-McAlphine, 2020 [115]	Nutrition and birth outcomes	Queensland	Ethnicity and/or COB or indigenous Australian	Cross-sectional study	Self-reported Survey	431
Kwan, 2019 [116]	Depression in dialysis patients	NSW	LSAH and aboriginal background	Cross-sectional study	Survey	110
Kippin, 2018 [117]	Young offender	Western Australia	LSAH/indigenous status	Cross-sectional study	Self-reported Survey	98
Shepherd, 2018 [118]	Young offender	Victoria	Self-identified (1) CALD = non-Australian cultural background/ (2) indigenous status/ (3) English speaking background	Cross-sectional study	Face to face interview	212
Shepherd, 2015 [119]	Re-offending	Victoria	Self-identified (1) CALD = non-Australian cultural background/ (2) indigenous status/ (3) English-speaking background	Cross-sectional study	Face to face interview	207
Chan, 2018 [120]	Hiv/aids	NSW	Self-reported overseas born/LSAH is not English, length of stay if born overseas/indigenous status	Retrospective, non-consecutive audit	Patientregistration data	264
Porter, 2016 [121]	Cultural group of pregnant women	NSW	Two different definitions, including self-reported COB and CALD/ethnicity, were compared	Cross-sectional study	Survey	762
Petrov, 2017 [122]	Nursing home	Victoria	Born in NESC and/or have a preferred language other than English	Cross-sectional study	AIHW National Aged Care Data Clearinghouse	44,925

**Table 2:** CALD definitions used in the selected studies.

Definitions of CALD	*n*	%
Country of birth (COB)	33	30.6
Language spoken at home (LSAH)	21	19.4
Indigenous status	3	2.8
Ethnicity/cultural/self-defined CALD background	15	13.9
Migrants and refugees	5	4.6
Combination of two or more definitions	31	28.7
The minimum core set of CALD definition *	0	0.0
Total	108	100.0

* The combination of four variables in the minimum core set (COB, LSAH, indigenous status, and English proficiency) to measure the CALD status of study participants in data collection.

**Table 3:** CALD definitions by data sources.

Definitions of CALD	Administrative/Available Data		Self-Collected Data	
	*n*	%	*n*	%
Country of Birth (COB)	18	34.6	15	26.8
Language spoken at home (LSAH) other than English	8	15.4	13	23.2
Indigenous status	1	1.9	2	3.6
Ethnicity/cultural/CALD background	6	11.5	9	16.1
Migrants and refugees	0	0.0	5	8.9
Combination of two above or more	19	36.5	12	21.4
Total	52	100.0	56	100.0

## Data Availability

Data sharing not applicable.

## Abbreviations

AIHW	Australian Institute of Health and Welfare
BAEM	Black and Asia Ethnic Minority
BAME	Black and Asia Minor Ethnicity
CALD	Cultural and Linguistic Diversity, Culturally and Linguistically Diverse
COB	Country of Birth
LSAH	Language spoken at home
NESC	Non-English-speaking countries
NSW	New South Wales
UNDRIP	United Nations Declaration on the Rights of Indigenous Peoples
